# Reduction of Elevated Proton Leak Rejuvenates Mitochondria in the Aged Cardiomyocyte

**DOI:** 10.1093/geroni/igaa057.1691

**Published:** 2020-12-16

**Authors:** Huiliang Zhang, Nathan Alder, Wang Wang, Hazel Szeto, David Marcinek, Peter Rabinovitch

**Affiliations:** 1 University of Washington, Seattle, Washington, United States; 2 University of Connecticut, Mansfield, Connecticut, United States; 3 Alexandria LaunchLabs, New York, New York, United States

## Abstract

Rational: Aging-associated diseases, including cardiac dysfunction, are increasingly common in the population. However, the mechanisms of physiologic aging in general, and cardiac aging in particular, remain poorly understood. While effective medical interventions are available for some kinds of heart failure, one age-related impairment, diastolic dysfunction in Heart Failure with Preserved Ejection Fraction (HFpEF) is lacking a clinically effective treatment. Methods and Results: Using the pH indicator cpYFP in the model of naturally aging mice and rats, we show direct evidence of increased mitochondrial proton leak in aged heart mitochondria following a pH gradient stress. Furthermore, we identified Adenine Nucleotide Translocator 1 (ANT1) as mediating the increased proton permeability of old cardiomyocytes. Most importantly, acute (2 hours) in vitro treatment with the tetra-peptide drug SS-31 (elamipretide) reverses age-related excess proton entry, decreases the mitochondrial flash activity and mitochondrial permeability transition pore (mPTP) opening and rejuvenates mitochondrial function. Moreover, we show that SS-31 benefits the old mitochondria by direct association with ANT1 and stabilization of the mitochondrial ATP synthasome, leading to substantial reversal of diastolic dysfunction. Conclusion: Our results uncover excessive mitochondrial proton leak as a novel mechanism of age-related cardiac dysfunction and elucidate how SS-31 is able to reverse this clinically important complication of cardiac aging.

